# SARS Risk Perception, Knowledge, Precautions, and Information Sources, the Netherlands

**DOI:** 10.3201/eid1008.040283

**Published:** 2004-08

**Authors:** Johannes Brug, Arja R. Aro, Anke Oenema, Onno de Zwart, Jan Hendrik Richardus, George D. Bishop

**Affiliations:** * Erasmus University Medical Center, Rotterdam, the Netherlands;; † National Public Health Institute, Helsinki, Finland;; ‡ Municipal Health Service of Rotterdam, Rotterdam, the Netherlands;; § National University of Singapore, Singapore

**Keywords:** SARS, knowledge, risk perceptions, precautionary behavior, information sources, dispatch

## Abstract

Severe acute respiratory syndrome (SARS)–related risk perceptions, knowledge, precautionary actions, and information sources were studied in the Netherlands during the 2003 SARS outbreak. Although respondents were highly aware of the SARS outbreak, the outbreak did not result in unnecessary precautionary actions or fears.

Severe acute respiratory syndrome (SARS) is one of the latest examples of an emerging infectious disease confronting the world (1). Outbreaks of diseases like SARS are expected to recur, and they may rapidly spread across the globe. Measures to control outbreaks include not only identifying new organisms, developing vaccines, and initiating appropriate therapies, but also adequately informing the public about risks and precautions. In an unaffected country like the Netherlands, true risk may have been low, but SARS still received broad media attention, which may have increased perception of risk. Perceived risk, not actual risk, determines the population's reaction (2,3), even though these perceptions are often biased (3). The public may be optimistic when familiar risks are perceived to be largely under volitional control; pessimism, sometimes leading to mass panic, is more likely a result of perceiving risks to be uncontrollable (2–5). Persons who perceive themselves to be at risk for SARS may engage in precautionary behavior, but they may also stigmatize those who are perceived as possible sources for infection (6). To promote realistic risk perceptions and effective precautions, communication through various information sources is essential (7,8).

## The Study

We explored SARS-related risk perceptions, knowledge, actions, and use of information sources in an area where no cases occurred during the 2003 SARS outbreak. Respondents were drawn from a random sample of 500 members of an Internet research panel who completed an electronic questionnaire on a Web site June 19–26, 2003. Respondents were 373 persons ages 19–78 years; 48% were male. Of the respondents, 37.2% had a low level of vocational or secondary education; 39.6% had an intermediate level of vocational or secondary education; 21.5% had professional or university training; and the remainder were missing values.

Data were collected with an electronic questionnaire developed by the SARS Psychosocial Research Consortium (G.D. Bishop et al., unpub. data; full questionnaire is available from http://www.eur.nl/fgg/mgz/papers.html). Risk perceptions were obtained by asking respondents how they estimated their risk of acquiring and dying from SARS. To compare the SARS-related risk perceptions to other potential threats, respondents were asked to indicate how likely they thought it was for them to get other diseases or have accidents (Table 1). Respondents were also asked how worried they were about contracting SARS, a family member getting SARS, SARS occurring in their region, SARS emerging as a health problem, and the likelihood of other persons acquiring SARS.

**Table 1:** Perceived risk of being affected by SARS and other diseases or accidents^a^

	Mean (SD)	% likely or very likely
SARS	1.5 (0.8)	1.0
Flu or common cold	4.0 (1.0)	72.9
Accident at home	3.5(1.0)	52.0
Cancer	3.0 (1.0)	18.5
Heart attack	2.9 (0.9)	21.7
Traffic accident	2.8 (0.9)	16.1
Food poisoning	2.8 (1.0)	21.4
HIV/AIDS	1.5 (1.9)	1.9

^a^SARS, severe acute respiratory syndrome; for the scores, 1 = very unlikely and 5 = very likely.

Knowledge about SARS was assessed with four questions on whether respondents had ever heard of SARS, knew what SARS is, knew its causes, and knew the death rate for people with the condition. A total SARS-related knowledge score was computed by adding the correct answers to the questions (range 0–4).

Respondents were asked whether they felt able to avoid contracting SARS and which actions they had taken to avoid getting it (Table 2). The total number of actions taken was regarded as an overall SARS precautionary behavior score (range 0–19, α = 0.72). Diagnostic actions that could be indicated included taking one's temperature; going to a physician; paying attention to coughing, sneezing, feelings of fatigue, and headaches; and calling a SARS hotline. The total number of actions was regarded as a diagnostic behavior score (range 0–8, α = 0.77). Respondents were asked to indicate how likely they were to avoid different persons to prevent SARS. Finally, respondents were asked to indicate how much information about SARS they obtained from different sources and how much confidence they had in these sources (Table 3).

**Table 2:** Proportion of respondents (N = 373) who reported specific actions to prevent severe acute respiratory syndrome (SARS)

Precautionary action	Percentage
Avoided travel to SARS-infected areas	39.9
Made sure to get sufficient sleep	8.3
Wore a mask	3.8
Avoided eating in "food centers"	2.9
Took an herbal supplement	2.4
Avoided large gatherings of people	2.1
Washed hands more often	2.1
Used disinfectants	2.1
Were more attentive to cleanliness	1.9
Avoided particular types of people	1.6
Ate a balanced diet	1.6
Avoided travel by airplane	1.1
Did not go to school or work	1.1
Avoided shaking hands	1.1
Avoided travel by taxis	0.5
Avoided travel on subways or buses	0.3
Avoided eating in restaurants	0.3
Exercised regularly	0.3

**Table 3:** Sources of information about severe acute respiratory syndrome (SARS) and confidence in those sources^a^

Information source	Amount of information, mean (95% CI)	Confidence in the information, mean (95% CI)
Television	3.9 (3.8–4.0)	3.6 (3.5–3.7)
Newspapers	3.5 (3.3–3.6)	3.4 (3.3–3.5)
Internet	2.3 (2.2–2.5)	3.0 (2.9–3.1)
Magazines	2.1 (2.0–2.3)	2.7 (2.6–2.8)
Health officials	1.7 (1.6–1.8)	3.3 (3.2–3.5)
Friends	1.6 (1.5–1.7)	2.5 (2.3–3.6)
Physicians	1.3 (1.2–1.4)	3.2 (3.1–3.4)

^a^Scale ranged from 1 = very little to 5 = very much. CI, confidence interval.

## Results

All but two of the respondents had heard of SARS. Most respondents knew that it is a severe type of pneumonia (91.2%) and caused by a virus (88.7%). The correct estimate of 15% for the death rate for SARS-infected patients was reported by 9%, while 34.1% made estimates close to that number (10%–20%). Equal proportions of the respondents underestimated (44.5%) and overestimated (46.4%) the death rate. A mean knowledge score of 2.9 (standard deviation [SD] = 0.5) was observed; 83.9% of the respondents answered three or more knowledge questions correctly.

While 38.9% were worried about SARS as a health problem, few respondents were worried about getting SARS themselves (4.9%), about family members acquiring it (8.3%), or about SARS in the Netherlands (4.9%). Only 2.6% rated their risk of getting SARS as high or very high; 1.6% thought it likely or very likely that they might die from SARS. The perceived likelihood for getting SARS was lower than for getting a heart attack and cancer but comparable to that for HIV/AIDS (Table 1). Thirty-three percent of respondents thought that their risk for SARS was lower than that for other persons of the same sex and age; 7.7% perceived their risk to be higher than that of others.

Perceived capability to avoid SARS was rated as good or very good by 40.5%; 12.3% rated their capability as poor or very poor. All respondents reported taking at least one precautionary action; 41.3% reported one or more specific actions, especially avoiding travel to a SARS-endemic area; the other respondents indicated they had done "something else" to avoid getting SARS (Table 2). A mean score of 2.9 (SD = 0.5) was obtained for precautionary actions.

Substantial proportions of respondents reported that they would avoid persons from a SARS-endemic area (50.0%), a person who has a family member with SARS (46.1%), persons possibly from a SARS-endemic area (27.8%), and strangers wearing a protective mask (31.9%). A few respondents (<7%) reported they would avoid healthcare workers or persons who had a cough, looked unwell, had a fever, or sneezed.

SARS diagnostic behavior was rare, with "paying close attention to coughing" (3.5%) reported most often. Only 2.7% had visited a doctor because of SARS-related worries, and 1.1% had called a SARS information telephone service. The mean score for diagnostic action was 0.1 (SD = 0.6).

Pearson correlations indicated that perceived risk of acquiring SARS was positively associated with worries and self-reported precautionary actions to avoid SARS, while negative associations were found with perceived ability to avoid SARS. Precautionary action to avoid SARS was further associated with worries related to the syndrome, and knowledge about SARS was associated with worries about the condition as a health problem (Table 4).

**Table 4:** Pearson correlations between severe acute respiratory syndrome (SARS)-related risk perceptions, knowledge, and actions

	1	2	3	4	5	6	7
1. Perceived risk of acquiring SARS							
2. Perceived risk of acquiring SARS compared to others	0.43^a^						
3. Worry about getting SARS	0.64^a^	0.31^a^					
4. Worry about SARS as a health problem	0.40^a^	0.34^a^	0.45^a^				
5. Knowledge about SARS	–0.10	0.02	–0.05	–0.02^b^			
6. Self-reported precautionary actions to avoid SARS	0.16^c^	0.05	0.23^a^	0.10	0.00		
7. Perceived ability to avoid SARS	–0.33^a^	–0.27^c^	–0.30^a^	–0.22^a^	–0.03	0.04	
8. Perceived ability to avoid SARS compared to others	–0.27^a^	–0.49^c^	–0.23^a^	–0.21^a^	–0.09	–0.03	0.30^a^

^a^p < 0.001. ^b^p < 0.05. ^c^p < 0.01.

Multiple linear regression analyses with SARS-related risk perceptions and worries as dependent variables and sex, age, and education as independent variables showed a significant association between sex and risk perceptions (standardized regression coefficient [β] = 0.23, p = 0.005) and between years of education and worries (β = –0.18, p = 0.007). Women perceived their risk as higher than men, and less educated persons were more worried about SARS than those with more years of education. No significant associations were found in regression analyses with precautionary actions or SARS-related knowledge as dependent variables.

## Conclusions

This study is the first to report on public perceptions of SARS outside the affected area. The results indicate that the Dutch population was well aware of the SARS outbreak, knew what SARS was, was not overly concerned about their risk, and obtained their information primarily from television and newspapers, which were also rated as trustworthy sources of information. Many respondents reported that they took precautionary actions to reduce their risk for SARS, but very few took possible diagnostic actions.

The present study builds upon earlier work from the SARS Psychosocial Research Consortium (G.D. Bishop et al., unpub. data). In that study, more respondents underestimated the death rate of SARS patients than in the present study (71% vs. 45%), with no significant difference between affected and unaffected countries. Our study was conducted later, which may have meant that more knowledge about SARS was available. Earlier studies (9,10) have reported on SARS-related risk perceptions during the outbreak in Hong Kong, and these studies reported quite different perceptions of high personal risk, ranging from 9%–30%. In our study, the perceived likelihood of getting SARS was rated high by few persons. Women reported higher perceptions of risk than men, and people with less education expressed more worries about the disease. Earlier studies on different topics reported mixed findings on differences in risk perceptions according to level of education (11–13). Higher perceptions of risk were associated with more worry and more self-reported precautionary actions, which is in line with predictions from risk perception theory and previous research (2,9). Avoiding air travel was the only precautionary action that was mentioned relatively often.

We conclude that the 2003 SARS outbreak did not lead to unwarranted precautionary actions or fears. Even though no SARS cases were discovered in the Netherlands, the Dutch population was well aware of the outbreak and was well informed about SARS, primarily through television and newspapers. The methods and results of the present study can be used for risk perception research during new outbreaks of SARS or other emerging infectious diseases.

Dr. Brug is professor of determinants of public health in the Department of Public Health, Erasmus University Medical Center, Rotterdam, the Netherlands. His research is focused on determinants of health-related behaviors and effectiveness of prevention interventions.
